# Ecotherapy – A Forgotten Ecosystem Service: A Review

**DOI:** 10.3389/fpsyg.2018.01389

**Published:** 2018-08-03

**Authors:** James K. Summers, Deborah N. Vivian

**Affiliations:** Gulf Ecology Division, National Health and Environmental Effects Research Laboratory, Office of Research and Development, United States Environmental Protection Agency, Gulf Breeze, FL, United States

**Keywords:** ecotherapy, ecosystem services, recovery times, nature, broaden-and-build theory

## Abstract

Natural ecosystems provide important services upon which humans depend. Unfortunately, some people tend to believe that these services are provided by nature for free; therefore, the services have little or no value. One nearly forgotten ecosystem service is ecotherapy – the ability of interaction with nature to enhance healing and growth. While we do not pay for this service, its loss can result in a cost to humans resulting in slower recovery times, greater distress and reduced well-being. Losses in these images of nature can diminish our basic happiness. Little is understood or, at least, appreciated concerning the potential ecotherapy benefits of the natural environment and its ecosystem services. The complex and interactive relationship of ecosystems, their services and human well-being is poorly acknowledged in the broad social, philosophical, psychological and economic well-being literature. In this article, we examine the role of nature and its ecosystem services in ecotherapy and its associated enhancement of recovery from physical and mental illness through a review of studies evaluating this ecosystem service-recovery connection.

## Introduction

Ecosystems provide basic services upon which humans depend. Unfortunately, people tend to believe that these ecosystem services are provided for free; therefore, the services are of little or no economic value. These services may not have a specific cost in dollars, but ordinary decisions by communities usually have an effect on the quality and magnitude of nature’s provided ecosystem services. While humans do not pay directly for them, we bear the significant cost for their loss regarding increased illness, reduced soil fertility, moratoriums on greenhouse gasses, wastewater treatments facilities, and losses in those images of natural ecosystems that enrich our basic happiness.

The entire human economy depends on the goods and services provided by natural ecosystems (Daily, 1997). The natural processes of restoration (cleaning, recharging, and recycling), along with goods such as forage, timber and seafood, are worth trillions of dollars annually. Nothing could survive without these ecosystem services. Growing human interventions on the environment can significantly alter the functioning of natural ecosystems reducing the delivery of their services. Ecosystems have been changed by humans more extensively and rapidly in the last 75 years than in any previous period of human existence (Daily, 1997). We have used these resources to meet the world’s growing demands for fiber, food, fuel, freshwater and timber. These alterations to ecosystems likely appear to raise the well-being of billions of people. However, these changes may have, unintentionally:

• Caused a major and sometimes largely permanent loss of biodiversity,
• Stressed the ability of natural systems to continue contributing necessary and important services,
• Aaltered our comfort level with nature and our sense of place and,
• Reduced human well-being significantly.

Ecotherapy is one of ecosystem services that nature provides and is based on the theories of ecopsychology. Broadly speaking it is an area of psychology that embraces ecology and aims to be holistic in theory and practice (Buzzell and Chalquist, 2009). This means that from an ecotherapy perspective, the health (physical and mental) of a human being is viewed in the context of the health of the Earth and its natural ecosystems (Swimme and Berry, 1994; Clinebell, 1996). Ecotherapy helps people connect with nature to aid in dealing with physical and mental illnesses (Buzzell and Chalquist, 2009). This idea of reconnection seeks to remind humans that we are part of ecosystems rather than separate from them (Jones, 2010; Totton, 2011). The philosophical approach is similar to philosophies of deep ecology known as ecosophy T (Naess, 1973, 1990, 2001). Ecotherapy is evidenced by numerous approaches – green exercise (Pretty et al., 2005, 2007), green views (Ulrich, 1984; de Vries et al., 2003), horticultural therapy (Linden and Grut, 2002), wilderness therapy (Russell, 2001), body therapy through movement (Clinebell, 1996), art therapy (Degges-White and Davis, 2010) and animal-assisted therapy (DeMayo, 2009). Sometimes, ecotherapy can be just taking more traditional talk therapy outside into a garden, public space, forest or beach. Ecotherapy often incorporates elements of mindfulness practices (Ambrose-Oji, 2013; Jordan and Hinds, 2016). During outdoor therapy, both nature and human beings serve as therapists, assisting the client toward healing.

Since the advent of major technological advancements, Western society has retreated from the “Great Outdoors” and placed more emphasis on technology; such as, television, computers, and gaming (Hartig et al., 2014; Chawla, 2015). Mounting evidence suggests that people, by pushing away from nature, have distanced themselves from major environmental issues (e.g., acute weather events, water quality, air quality) and, in the end, have begun to lose contact with a necessary tool for their mental health that is available to all at little or no cost. By denying interactions with natural ecosystems, people jeopardize the rejection of a basic part of our being – a principle that is ironically more evident due to advances in medical technology (van den Berg et al., 2010; Thompson et al., 2012).

Healers in many medical systems, from Ayurvedic medicine (Chopra and Doiphode, 2002) to traditional Chinese medicine (Kayne and Booker, 2010) to many Western pediatric perspectives (Little and Wyver, 2008; Prince et al., 2013), have long advocated the importance of nature to well-being. However, the concept that flowers and trees can influence well-being, psychologically, was largely untested until the late 1970s, when R.S. Ulrich examined the psychological influence of scenes of nature on stress experienced by students (Ulrich, 1979, 1981, 1983, 1986) and medical recovery rates (Ulrich, 1984). His testing showed changes in mental states and conditions after students observed “natural” scenes associated with the environment. These scenes increased positive feelings of friendliness, affection, joy and playfulness. Views of non-nature based phenomena like urban settings, on the other hand, resulted significantly in one primary feeling: sadness. Viewing urban scenes also had a tendency to increase feelings of aggression and anger while viewing nature tended to reduce those feelings. Scenes of nature and natural ecosystems fostered positive thoughts and lowered anger and aggression. Based on these findings, Ulrich measured brain activity in healthy, unstressed adults and demonstrated that seeing landscapes associated with nature resulted in the increased production of serotonin (Ulrich et al., 1991). Many antidepressant medications used in Western medicine are thought to work by elevating the availability of serotonin to enhance communication among nerve cells. Many subsequent researchers have conducted objective testing to confirm this phenomenon. Ulrich’s pioneering research showing changes in surgery recovery times based on patients’ window views of nature (trees) and urban scenes (walls, concrete) demonstrated this “natural” capacity extended beyond feelings to detectable medical phenomena.

Nature, whether you’re in the woods far away from it all, in a city park, or simply walking down a tree-lined street, has the power to make people feel new again. Studies have shown that a simple walk in nature can reduce anxiety, keep your spirits high, and even improve memory. Even just looking at photographs of greenery for less than a minute can give you a mood boost. Spending time in nature reduces stress and helps people feel energetic and more alive, according to scientists at the University of Rochester (Brown and Ryan, 2003). A recent study used mobile EEG devices to monitor participants’ emotions during a walk in nature. Researchers also found that people were more likely to experience meditative-like brain waves and exhibit less frustration if they were walking in a green space, compared to a bustling shopping street or a busy business area (Aspinall et al., 2013).

Greencare is recognized as an increasingly important phenomenon. It encompasses or involves activities such as care farming, animal-assisted interventions (AAI), social and therapeutic horticulture (STH), healing gardens and facilitated green exercise. Despite the importance of Greencare therapies, there is a lack of appreciation that all of these care intervention types and related research are the result of a simple ecosystem service. Humans’ need for nature is more than a simple requirement for material exploitation. Humans also need interaction with nature and its ecosystems to enhance our cognitive, emotional, spiritual and aesthetic development. This review will examine the role of this important ecosystem service (Nature being there) in therapies for several disorders and for several developmental aspects. These include the following physical and mental health disorders:

• General medical recovery (e.g., heart rate, blood pressure, surgery recovery, cardiopulmonary rehabilitation)
• Pain reduction
• Mood and Stress (e.g., post-traumatic stress, anxiety, self-esteem, addiction, mental well-being)
• Attention Deficit/Hyperactivity Disorder
• Dementia
• Obesity
• Other Disorders (e.g., vitamin D deficiencies, general mental health issues)

Finally, Nature therapy is important for several normal developmental aspects of children and the maintenance of those aspects for adults. Therefore, the following developmental aspects are considered in this review:

• Creativity
• Cognition
• Restoration
• Well-being and Life Satisfaction.

## Physical and Mental Health Disorders

### Medical Recovery

One of the first observations of the restorative effects of nature in a medical setting showed more rapid recovery rates from gall bladder surgery if patients had a view of nature through their windows versus either no window or no natural view (Ulrich, 1984). Anxiety was reduced in these patients and the recovery times of patients with a “view” of nature was half that of those with a view of a wall. Ulrich also measured brain activity in healthy, unstressed adults and demonstrated that viewing scenes of nature was associated with the elevated production of serotonin (Ulrich et al., 1991). Viewing nature scenes stimulated positive thinking and reduced aggression and post-stress anger. Ulrich’s pioneering research showing changes in recovery times following operations based on patients’ window views of nature (trees) and urban scenes (walls, concrete) demonstrated this “natural” capacity extended beyond feelings to tangible medical phenomena. Many other researchers have used objective testing to confirm this phenomenon.

Flowering plants and foliage in hospital rooms have attributed to enhanced recovery rates of patients undergoing appendectomies (Park and Mattson, 2008, 2009a). Patients in rooms with flowers and plants required less post-operative medications, demonstrated more positive physiological responses (heart rate, anxiety and fatigue, lower systolic blood pressure, pain ratings) and had more positive emotions and greater satisfaction with their hospital rooms than those in the control group. Indoor ornamental plants were also linked to generalized enhanced health outcomes in patients recovering from surgery (Park and Mattson, 2009b). Indoor plant exposure in Norway enhanced mental health recovery rates of coronary and pulmonary patients but did not enhance their physical recovery (Raanaas et al., 2010). A recent review (Bringslimark et al., 2009) cataloged the numerous psychological benefits of passive indoor plant exposures. Passive exposure results are mixed but plant exposure has been shown to result in a variety of outcomes, including reduced pain perception, enhanced emotional states, reduced autonomic arousal, and enhanced creativity and task-performance.

### Pain Reduction

There has recently been a heightened recognition that environmental factors, including exposure to nature scenes, can influence pain (Malenbaum et al., 2008). Wilson (1984) suggested that human beings have an inherent bond with nature and the contact with the natural world could be beneficial to human health. Given this connection, it is reasonable that nature, natural settings and plants could be useful in healthcare facility design targeted to reduce pain. Natural views of landscapes are not always accessible for hospitalized patients but, even, using simple images of nature enhance recovery rates and pain reduction of coronary surgical patients (Ulrich et al., 1993). Patients exposed to images of nature were much more likely to change from stronger to weaker pain medication during recovery. Patients exposed to nature images reported significantly less anxiety as well.

Combining nature sounds and images was shown to reduce pain in a randomized clinical trial of patients undergoing flexible bronchoscopies (Diette et al., 2003). Patients who were exposed to sounds and scenes of nature reported significantly enhanced levels of perceived pain control. In an experiment where healthy participants had pain induced, exposure to a video of natural scenery increased pain tolerance and threshold (Tse et al., 2002). Exposure of increased levels of sunlight for patients having undergone spinal surgery resulted in reduced pain, stress, use of painkilling medication and the overall costs of pain medication (Walch et al., 2005).

Biomonitoring experimental sessions showed increases in pain tolerance as a result of exposure to ornamental plants in a simulated hospital room (Park et al., 2002). Similarly, pain perception appears to be altered by exposure to nature (Lohr and Pearson-Mims, 2000). Subjects were more willing to keep a hand submerged in ice water for 5 min if they were in a room with flowers than in a room without plants. However, this “plant” effect was also observed when subjects in non-plant rooms were provided other “non-nature” stimuli to distract them (e.g., bright colors).

### PTSD, Mood Modification and Stress Reduction

Post-Traumatic Stress Disorder (PTSD) is one of the most compelling costs of war. PTSD can be defined as an anxiety that can develop after exposure to a terrifying event in which grave physical harm occurred or was threatened (NIMH, 2011). The prevalence of PTSD among veterans has been pronounced over the years, ranging from about 30% for men and women during the Vietnam era (Kulka et al., 1990) to 12% in the Gulf War (Kang et al., 2003) to about 23% overall in Afghanistan/Iraq conflicts (Tanielian and Jaycox, 2008; Ramchand et al., 2010; Fulton et al., 2015). Typical mental health treatments for these veterans include trauma-focused cognitive behavioral therapies (e.g., cognitive-processing therapy, cognitive restructuring, exposure therapy, stress inoculation therapy) (Taylor et al., 2003; Hassija and Gray, 2010; Hoge, 2011), eye movement desensitization and reprocessing (Macklin et al., 2000; Shapiro, 2014) and pharmaceuticals such as selective serotonin uptake inhibitors (Marshall et al., 2001; Stein et al., 2002; Hoge, 2011).

An alternative strengths-based strategy for PTSD treatment has been various forms of recreation-based ecotherapy (Hawkins et al., 2016). Strength-based approaches focus on internal strengths (e.g., interests, beliefs, talent abilities, skills, knowledge, aspirations, character strengths, virtues), external strengths (e.g., family support and involvement, social support, positive attitudes, community and home resources, ecological factors) and existing skill sets (e.g., character strengths, military skill sets). The individual’s hopes, aspirations and values take priority in treatment instead of medically directed care that focuses on reducing symptoms and functional deficits (Anderson and Heyne, 2012; Heyne and Anderson, 2012). Based on Attention-Restoration Theory (ART, Kaplan and Kaplan, 1989), this type of strengths-based therapy proposes that people are restored in natural environments because they escape from usual settings and become fascinated by stimulation in natural ecosystems that take their mind off their day-to-day problems. Outdoor adventure, wilderness therapy, outdoor experience and green space-based ecotherapy (e.g., whitewater river rafting, fly-fishing, educational decision-making in nature, interactions and participation in nature) have been shown to be effective therapeutic media for veterans coping with PTSD (Berman and Davis-Berman, 1995; Hattie et al., 1997; Fredrickson and Anderson, 1999; Ewert et al., 2001; Burls, 2007; Dustin et al., 2011; Mowatt and Bennett, 2011; Sibthorp and Jostad, 2014). For many veterans, being in nature is emotionally calming and helps them manage negative mental health symptoms through immersion in novel, natural environments. As a result of ecotherapy, many veterans can see beyond their past military experiences and injuries and establish a greater sense of purpose beyond themselves.

Green space and wilderness therapy are two ecotherapy approaches being used to address mood modification and stress reduction. Green space is important for physical and mental well-being. Interaction and engagement with green space have been linked with increased length of life and deceased risk of mental illness across a number of countries (Takano et al., 2002). Wilderness therapy is a treatment which uses a structured approach to work with adolescents with behavioral problems (Russell et al., 1999; Hill, 2007). This type of therapy is most frequently used with adolescents at risk to help them deal with a variety of psychological problems such as adjustment, emotional or addiction (Annerstedt and Wahrborg, 2011). The mental health conditions that can be addressed by these types of ecotherapy include anxiety, depression, self-esteem, addiction and stress reduction.

Coronary heart disease patients are often offered some form of rehabilitation that generally involves a combination of health education and exercise. Psychosocial mediations aimed at reducing such risks factors as anxiety and stress are less regularly included although a large body of work indicates they can be successful in modifying the progression of coronary heart disease (Ornish et al., 1990; Krantz and McCeney, 2002). Following a myocardial event, cardiac patients report high levels of anxiety and stress during hospitalization and post-discharge. A patient’s overall mood can modify rehabilitative efforts. An affirmative emotional state can offer people the freedom to examine plans for the future. Gardening is a popular and often available method of recreational ecotherapy that lends itself to a healthy lifestyle. Horticultural therapy (HT) is a process through which gardening activities, interaction with plants and closeness to nature are used as a rehabilitative strategy (Simson and Straus, 1998). Horticultural therapy has been shown to improve mood state reducing stress and its contribution to coronary heart disease (based on POMS score) (Wichrowski et al., 2005), improve self-esteem and reduce depression (Son et al., 2004; Lee et al., 2008), improve sleep and cognitive issues in dementia patients (Lee and Kim, 2008), improve engagement and mood-related to dementia (Gigliotti et al., 2004; Gigliotti and Jarrott, 2005) and as a general treatment for mental health issues (Szofran and Myer, 2004). Further, horticultural activities (Richards and Kafami, 1999) and integrated adventure therapy programs (Bennett et al., 1998), have been shown to be useful in substance abuse treatment.

Adventure-based treatment programs have shown success in treating self-esteem issues, schizophrenia, mood modification, adolescent behavior, school success, anger management, sociality and family functionality (Wilson and Lipsey, 2000). Adventure- and recreation-based group interventions have been useful in promoting well-being and weight loss in schizophrenia (Voruganti et al., 2006). A 2-week wilderness camp enhanced 10 community-level coping skills related to community survival of chronic mentally ill patients (Banaka and Young, 1985). Participation in a 10-day winter outdoor adventure enhanced the self-concept and locus of control for hearing-impaired individuals (Luckner, 1989a,b). Similarly, outdoor experiential approaches have proven useful in promoting adjustment to brain injury (Thomas, 2004). One of the most useful applications of wilderness and outdoor experiences has been with the improvement of family functionality and well-being (Davis-Berman and Berman, 1989; Harper and Cooley, 2007; Harper et al., 2007; Harper and Russell, 2008), adolescent attachment (Bettman, 2007) and chemical dependency (Kennedy and Minami, 1993).

Healing gardens and natural ecosystem encounters have been shown to reduce depression (McCaffrey, 2007), restore attention in cancer patients (Cimprich and Ronis, 2003), treat dementia (Detweiler et al., 2008) and reduce stress (Kohlleppel and Bradley, 2002). Wells and Evans (2003) reported that 8–10 year-old children from rural areas who were exposed to high levels of nearby nature experienced less stress and tended to recover from stress events more rapidly than children living in homes that lacked direct contact with nature. Cause and effect are difficult to disentangle in these interactions – does nature provide an opportunity for stress recovery; or does contact with nature assist in the development of coping mechanisms; or does it enhance possibilities for interaction with other children; or is the improved stress tolerance simply due to a combination of social and environmental factors? Almost twice as many children chose to play in spaces with trees than in spaces lacking natural elements (Taylor et al., 1998).

### ADHD

The lack of contact with nature (Louv, 2008) has been suggested to be one of the primary reasons underlying the recent surge in childhood maladies like Attention Deficit Hyperactivity Disorder (ADHD) (van der Berg and van der Berg, 2010). Over 6 million children in the United States are struggling to cope with chronic attentional deficit or attention-deficit/hyperactivity disorder (ADHD) (CDC, 2017a). ADHD reduces children’s attentional capacity and can have detrimental effects on many aspects of their lives (e.g., interpersonal relationships, school, personal growth). Many current treatments for ADHD have limited success and have numerous weaknesses, including appetite suppression, sleep disruption, depression and flattened affect (Douglas, 1972; Fiore et al., 1993; Hinshaw, 1994; Smucker and Hedayat, 2001; Purdie et al., 2002; Collingwood, 2010). Similarly, behavioral therapies, the second form of ADHD treatment (e.g., direct contingency management, self-monitoring), are typically insufficient to bring children into normal ranges of functioning (Hinshaw, 1994). Unfortunately, some available treatments have costly side effects and many have limited effectiveness. Attention Restoration Theory proposes that contact with nature and natural ecosystems support attention enhancement and many studies have demonstrated that contact with nature can result in increased attention in adults (Kaplan, 1995) and children (Taylor et al., 2001).

Factors like children’s motor ability, concentration and social play are all positively influenced following interaction or play in nature (Fjortoft and Sageie, 2000; Fjortoft, 2001, 2004). This improvement is particularly apparent involving children with ADHD (Taylor et al., 2001; Kuo and Taylor, 2004; Taylor and Kuo, 2009). Exposure to an ordinary natural setting (i.e., Nature) may be widely effective in reducing attention deficit symptoms in children. Increased green outdoors activities result in reduced children’s ADHD symptoms and have more positive affect effects on symptoms than activities in other settings (Kuo and Taylor, 2004). This green advantage was found among children who lived in a variety of community types regardless of community size, geographic region or household income. This positive effect of natural exposure on ADHD symptoms cannot be the result of the novelty of exposure to green spaces for urban children as rural children show similar positive results (Kuo and Taylor, 2004).

Attention Restoration Theory (ART) (James, 1962; Kaplan, 1995) was originally developed in environmental psychology to explain why people consistently reported a sense of renewal after wilderness and other natural environment encounters. Adults and children tend to perform systematically better on objective attention measures after viewing or spending time in natural surroundings (Tennessen and Cimprich, 1995; Kuo, 2001; Taylor et al., 2002; Taylor and Kuo, 2009).

### Dementia

Nature-related activities are a normal part of life – pottering in a garden, looking out a window or walking in the countryside. Such basic pleasures are often unattainable for a person with dementia living in a care facility. Holistic, interdisciplinary approaches to integrating nature into dementia care facilities provide care that supports both natural sensory stimulation and nature-based activities (Chalfont, 2007). Horticultural therapy for dementia patients seeks to increase human contact while engaging clients with nature (Abbott et al., 1997; van Loon, 2004). The modification of dementia residential design plans in order to incorporate plants, nature and gardens have shown positive effects (Day et al., 2000; Cobley, 2002; Chalfont, 2005).

Agitated aggressive behavior often occurs in late stage dementia. This behavior usually results in the use of chemical and physical restraints which can have significant side effects. Environmental psychologists have shown that exposure to nature and natural settings decreases agitation (Whall et al., 1997). Walled gardens appear to have a positive effect on the morale of special care dementia patients but do not always result in reductions in disruptive behaviors (Lovering, 1990; Mather et al., 1997).

### Obesity

More than 36% of United States adults and 17% of United States children are classified as obese (CDC, 2017b) and the number is increasing annually. The medical cost of obesity in the United States alone is estimated to be over $150 billion (CDC, 2017b). Globally the obesity rate increases to about 50% for adults and is about the same for children (OECD, 2017). Common health consequences of obesity include cardiovascular diseases (mainly heart disease and stroke), musculoskeletal disorders (especially osteoarthritis), diabetes and some cancers (e.g., endometrial, breast, ovarian, prostate, liver, gall bladder, kidney and colon). Childhood obesity is associated with higher chance of adult obesity, premature death and disability in adulthood. In addition to the higher likelihood of these maladies in adulthood, obese children often experience difficulties in breathing, higher risk of fractures, insulin resistance, hypertension, early markers of cardiovascular disease and mental health issues.

The interaction between the children’s physical activity and the environment is very complex. Physical activity is important for children’s health at all ages. It is clear that physical activity is strongly related to both the obesity and fitness of children. Both obesity and fitness track into adulthood where they can enhance risk factors for cardiovascular disease, metabolic disorders and early mortality. People with ready access to nature are less likely to be obese, inactive or dependent on anti-depressants (Neslen, 2017). Greenspace is an important resource for physical activity. It has the potential to contribute to the reduction of obesity and to improve health. In a review of quantitative research examining the association of greenspace and physical activity, weight status and health condition related to elevated weight, the majority of studies found a positive, but weak, association between greenspace and obesity-related health indicators (Lachowycz and Jones, 2011). Increased vegetation and greenspace were reported to be associated with reduced weight (Liu et al., 2007; Tilt et al., 2007; Bell et al., 2008). In eight major European cities, people were 40% less likely to be obese in the greenest areas of those cities (Ellaway et al., 2005).

### Other Disorders (Vitamin D)

Exposure to the sun is a requirement for the synthesis of adequate amounts of vitamin D by humans. Ultraviolet B from sunlight is absorbed by dehydrocholesterol in the skin which is subsequently transformed and converted to vitamin D3. Then, the liver metabolizes the vitamin into its biologically active form. Lack of vitamin D is recognized as a potential cause of rickets in children and elevating the potential for osteoporosis and even osteomalacia in adults. Similarly, as a result of more recent findings, it has been recognized that deficiency of vitamin D is correlated with increased multiple sclerosis, cardiovascular disease, some cancers, type I diabetes and rheumatoid arthritis, with possible links to schizophrenia and type II diabetes (Holick, 2004).

Possibly due to overall reduction of sunlight exposure, people living at higher latitudes have reduced incidence of multiple sclerosis (MS) although Norway appears to be an exception. This Norwegian anomaly may be the result of the enhanced outdoor activities by children (Kampman et al., 2007). It is possible that concerns over skin cancers being related to extensive exposure to the sun in combination with people spending less time outdoors is reducing the general population’s exposure to sunlight resulting in a reduction the incidence of these chronic diseases.

## Development, Interaction With Nature and Restoration

Children, today, grow up with a variety of indoor play facilities to choose from, including videogames, indoor play gardens, television and even indoor playground equipment; (Karsten, 2005). Increasing urbanization has significantly reduced the opportunity for safe outdoor play in cities and, even, in the suburbs. In order to protect them from harm, many parents actively discourage children from going outdoors (Veitch et al., 2010). As a result, more children are growing up disconnected from nature and the outdoors. This severing from interactions with nature could have important ramifications for children’s well-being and healthy development (Little and Wyver, 2008).

### Self-Esteem, Creativity and Development

Researchers have established significant and strong connections between direct contacts with nature and strengthened development in children (Bandoroff and Schrer, 1994; Kellert and Derr, 1998; Kuo and Taylor, 2004; Noddings, 2006; Louv, 2008). Kellert (2002) concluded that direct contact with nature significantly and positively impacts children’s affective, cognitive, and moral development. Wells and Evans (2003) showed that scores for anxiety, behavioral conduct disorders and depression were lower for rural children living near nature. Children living near natural ecosystems rated themselves higher on measures of self-worth than their peers in less natural settings (Wells and Evans, 2003). The greener a child’s view from their apartment, the higher he or she scored on several measures of delay of gratification and impulse control (Taylor et al., 2002).

Children’s general access to nature seems to be diminishing (Kahn, 2002; Kellert, 2002). Not only is there less nature for children to access but many parents may be limiting children’s freedom to access nature for fear of violence and accident (Spencer and Wooley, 2000; Louv, 2008). Children’s lives are increasingly filled with programmed activities, leaving them with minimal time for exploring nature. A diverse literature has explored the potential impacts of green spaces on healthy child development. Some of the most exciting findings of a link between contact with nature and developmental outcomes in children come from the effects of outdoor challenge programs on children’s self-esteem and sense of self. These findings suggest that contact with nature is likely to have significant benefits for children’s development (Kaplan, 1977; Kaplan and Talbot, 1983; Kellert and Derr, 1998). Similarly, many studies suggested a systematic relationship between outdoor curricula in green space and enhanced learning (Basile, 2000; Ratanapojnard, 2001). Studies comparing creative play in natural versus built spaces are consistent with nature supporting cognitive, social and emotional development (Kirkby, 1989; Taylor et al., 1998).

While methodological arguments could be raised with several of the above studies, the patterns of findings point in the same direction and the persistence of findings across cultural groups and numerous childhood settings. The general belief that contact with nature is supportive in several domains of children’s development – cognitive, social and emotional. Just as children require good nutrition and sleep patterns for positive development, they also need contact with nature.

### Cognition

Research into childhood outdoor experiences has identified increased cognitive functioning to be a key benefit of interaction with ecosystems (Chipeniuk, 1995; Falk and Dierking, 1997; Wells, 2000; Kisiel, 2005; Tzoulas et al., 2007). In a longitudinal study of children in low-income families where the families were relocated to houses with more nearby nature, the children had higher levels of cognitive functioning and an enhanced ability to direct attention which continued several months after returning to their original homes (Wells, 2000).

### Restoration

Evidence pointing to the psychological and restorative benefits of nature has accumulated significantly over the past several decades. Olmsted (1865) was particularly sensitive to the role of nature (i.e., natural scenery) in restoration. The early writings of Thoreau and his perceptiveness and foresight are likely more appreciated today (Anderson, 1968; Stern, 1970). While these writings have great power and provide deep inspiration for some, the more empirical evidence is convincing for others. Several studies (Kaplan and Kaplan, 1989; Relf, 1992; Hartig et al., 2003; Berman et al., 2008; Bowler et al., 2010) have addressed the potential restorative qualities of the interaction with nature.

Studies in the 1990s demonstrated the restorative influence of interactions with nature with regard to directed attention (Hartig et al., 1991), information processing effectiveness (Hartig et al., 1991), cancer patient enhanced effectiveness recovery (Cimprich, 1992, 1993) and the restorative benefits of a natural view on attentiveness (Tennessen and Cimprich, 1995). These studies demonstrated there is a link between restorative experience and directed attention.

### Well-Being/Life Satisfaction

In recent years, interest has grown in the positive benefits that might be gained from natural ecosystems and time spent outdoors with regard to an individual’s well-being (Pretty et al., 2003, 2005, 2007; Bird, 2007; Burls, 2007; MIND, 2007; Peacock et al., 2007). Because many people live in towns and cities, there are a number of efforts, even including exercise, to reconnect people with nature. Participating in physical activity and experiencing nature both play an important role in positively influencing our health and well-being. Short-term walking interventions, particularly in greenspaces, energize and enhance personal well-being and vitality (Peacock et al., 2007; Plante et al., 2007; Teas et al., 2007; Barton et al., 2009; Focht, 2009; Ryan et al., 2010) although walking combined with virtual reality settings depicting natural ecosystems also relaxes and enhances well-being (Plante et al., 2003, 2006). Similarly, running in nature enhances the exercise experience, modifies physiology and mood and increases overall well-being (McMurray et al., 1988; Harte and Eifert, 1995; Kerr et al., 2006; Hug et al., 2008). Research has established a strong link between contact with nature and enhanced human well-being (Greenleaf et al., 2014).

## Discussion

The primary interest of this review is to bring attention to an ecosystem service that is often overlooked, particularly by ecosystem services researchers. These researchers primarily address issues associated with the cleansing of air and water, the recycling of nutrients, the decomposition of waste and the support of living natural resources used for food and fiber. Nature’s impact of human physical and mental health can be just as important a service to humans as the services listed above. However, in conducting this review, there are natural issues which arise outside of the ecosystem service’s identification. For example, how good is the information relating the impact of nature interactions on these human health conditions? Does it indicate a strong causal linkage or a more causal association? Similarly, what are the underlying psychological processes underlying these relationships? While not, the main intent of the review, a discussion following which addresses these two points – (1) potential underlying mechanisms for these phenomena and (2) associational versus causal evidence for these impacts.

The stress of an unpleasant environment can result in feeling anxious, sad, helpless or depressed. These negative emotions, in turn, elevate heart rate, blood pressure and muscle tension which can suppress the immune system (Numeroff, 1983). Pleasing environments (e.g., nature) seem to have a reverse effect (e.g., most of the literature cited in this review). Researchers don’t yet understand all the details of why changes like these occur, but one possible explanation is that the types of interaction with nature described in this review reduce stress (e.g., Kohlleppel and Bradley, 2002; Hartig et al., 2003; Wells and Evans, 2003) and help people develop a more positive outlook (Folkman, 2008) both of which have been shown to strengthen the body’s immune system (Dillon et al., 1986; Reiche et al., 2004; Segerstrom and Miller, 2004).

At the most basic level, the purpose of nature-based therapeutic programs is behavior change (Maller et al., 2006). This therapeutic approach focuses on the utility of positive emotions to combat the symptoms and basis of illness. The examination of positive emotions is this manner is relatively recent (Fredrickson, 1998, 2001). Positive emotions are any feeling where there is a lack of negativity. Fredrickson (2009) identifies the 10 most common positive emotions as joy, gratitude, serenity, interest, hope, pride, amusement, inspiration, awe and love. Fredrickson (2001) formulated a new theoretical psychological model to better capture the utility of positive emotion called Broaden-and-Build Theory. This theory is in contract to traditional psychological models which described the function of negative emotions and their relationships to psychological outcomes. Life threatening circumstances often result in quick and decisive actions that are linked to negative emotions.

Although positive emotions can occur in these types of negative situations, they generally occur in non-life-threatening circumstances. Interactions with nature support several of the key propositions of the broaden-and build theory and can enhance cognition as well as intrinsic motivation to attachment styles and behavior (Fredrickson, 1998). The creation of these distinct kinds of positive emotions broaden and individual’s short-term thought action processes – enhancing their abilities to cope or adjust to mental health and developmental situations. Interaction with nature develops these positive emotions and the use the connection forms the basis for eco-therapy (Buzzell and Chalquist, 2009).

Evidence (most of the studies cited in this review) suggests that enhancement of these positive emotions results in broadened scopes of cognition, attention and action; thus, addressing disorders like stress, PSTD, ADHD, and dementia. Similarly, increases in positive emotions promote well-being, sense of security, and connection to nature building intellectual, social and physical resources (Fredrickson and Branigan, 2005).

It is very difficult to “prove” or even effectively demonstrate causality in nature interaction studies involving humans. The human-nature interaction is often a holistic phenomenon not easily reduced to a reductionist hypothesis-testing approach holding only one factor in change versus a control where the factor is not changed and all other factors are held constant. Human interactions are holistic and not reductionist; therefore, several potential “causes” are always possible in many of the studies reviewed here. This likelihood of numerous “causes” often leads social scientists to evaluate using a weight of evidence approach (preponderance of associational relationships) rather than the typical hypothesis-testing approach of the natural scientist. Even many, natural scientists use observational finding (associations) to develop theoretical constructs addressing large holistic phenomena and then support these theories using reductionist experiments. Many, if not most, large-scale theoretical advances have been the result of holistic associational inferences based on associational data and then supplemented by hypothesis-testing experimentation where feasible. Such is often the case in studies of the impacts of interaction with nature on the human condition.

With this phenomenological tenet in mind, the previous discussed studies are assessed based on the nature of the relationships – associational, weight of evidence, holistic or causal. The intent of these comparisons is not to lessen the impact of associational studies but to assuage critics of non-hypothesis-testing results as being less persuasive that direct hypothesis-testing experimentation. Supplementary Table S1 provides an overview of the studies reviewed herein and categorizes their findings as associational, associational weight of evidence, implied causal based on holistic evidence, or causal based on quasi-rigorous or rigorous experimentation. Much of the studies relating the interaction with nature and positive physical and mental health. Of the 123 studies reviewed here, the large majority (85%) includes associational information based on observations and surveys as opposed to rigorous causal hypothesis testing. This use of associational relationships does not negate the potential of a relationship but simply suggests that other co-linear information may be confuse the specific likelihood of a specific mechanism being identified. In many instances, many of these associational relationships (30%) are the result of quasi-experimental designs that differentiate between dual or multiple groups for a specific factor but let all other factors vary as they simply occur (addressing that all other factors vary similarly). While associational information dominates the type of analyses in all types of studies linking interactions with nature and health impacts, this is the common approach used by social scientists, non-research health practitioners, and public health departments. The utility of these associations is make multiple observations to create a weight-of-evidence for a set of hypotheses relating nature and health outcomes.

While the social sciences tend to prefer the weight-of-evidence and associational approaches, 51% of the reviewed studies represented quasi-rigorously or rigorously designed hypothesis testing experiments to support the linkages between interactions with nature and changes in physical and mental health. This is a common approach for the medical research community (representing about a quarter on the hypothesis testing experiments). The fact that roughly half of the studies reviewed used some type of hypothesis-testing experimental design suggest that the linkages for some relationships – for example, nature views (including plant life) on medical recovery, pain reduction and pain tolerance; some wilderness challenges impacts on behavioral modifications and locus of control issues; some gardening and nature interactions effects on disease treatment and blood chemistry; and, nature exposure and outdoor exercise on stress reduction.

Most hypothesis-testing designs are also associational (by statistical design) so nearly 45% of the results of the reviewed studies were both hypothesis-testing and associational. The remaining 40% of associational results were largely linking recreation-based ecotherapy to changes in condition for PTSD patients; mood, self-worth and well-being modifications resulting from interactions with nature or greenspace; many behavioral modification in adolescents (including substance abuse) resulting from wilderness encounters; attention deficit improvements from nature encounters; impacts of nature encounters on dementia improvement; and nature interactions improvements to childhood development, coping skills, and cognition. Similarly, the connections between outdoor exercise and greenspaces with reduction in obesity were largely associational.

## Conclusion

Clear and abundant evidence demonstrates that interaction with nature affects not only well-being but health throughout life. The evidence suggests that people, who as children strongly interact with ecosystems and environment, live longer with a better quality of life. This “therapy” tends to make them more active, connected to people and society, engaged with natural places and eat healthier foods. These interactions, even as an adult, often result in lower blood C-reactive proteins and cortisol levels. As a result, children and adults who interact with nature and natural settings tend to be members of groups and volunteer more, have higher self-esteem and better mood, keep learning, and continue regularly to engage with nature and be more resilient to stress. Conversely, people, who, particularly as children, tended to stay indoors (and thus not receive this “therapy”), appear to be more inactive or sedentary, disconnected from society, eat energy-dense and unhealthy foods, and have higher levels of blood c-reactive proteins and cortisol.

This review has highlighted the role of ecosystems and human-ecosystem interaction as a therapeutic device for a variety of physical, mental and developmental health issues to develop:

• Mobility, dexterity, stamina and resilience;
• Relief of depression and anxiety and improved concentration and memory; and
• Self-management, improved social and familial relations and skills, and self-esteem.

The research and literature seem to support the theoretical benefits derived from ecotherapy and human-ecological interactions. Thus, it seems rather obvious that:

• Being in nature affects health (physical and mental) positively. Being able to regularly get away from your built environment (house or office) and perform activities in a natural setting (or just being able to rest in a natural setting) can restore mental state and physical capacities (Hartig, 2007; Bjork et al., 2008; Grahn et al., 2010);
• Nature affects health positively for most people (Ulrich, 1999, 2001) or some people based on the interaction (Grahn and Stigsdotter, 2010);
• Many nature-based activities affect health positively but may depend on the context of the surrounding environment (Burls, 2007, 2008; Ottoson and Grahn, 2008; Grahn et al., 2010); and
• Some people will be more affected than others by treatment in nature-based therapeutic settings (Grahn et al., 2010).

It seems clear that this service that Nature provides (e.g., Nature being there to provide therapeutic or developmental services), without direct cost, is an underappreciated, if not near-forgotten, ecosystem service in the ecological literature regarding intermediate and final ecosystem goods and services. While often overlooked, the Ecotherapy service provided by nature is a very meaningful and important ecosystem service, worthy of conservation and regulatory costs. In reality, these economic costs which would be more than offset by the costs of medication and treatment through more traditional medical therapies. This discussion of the need and costs of preservation of natural ecosystems, if only for their therapeutic advantages, provides a substantive example of the enhancement of well-being through holistic discourse compared to the less than holistic small talk conversations concerning the continuing development of natural ecosystems strictly for economic growth (Mehl et al., 2010).

## Author Contributions

JS was responsible for the bulk of this review manuscript including writing, assessment, and evaluation of materials. DV was responsible for the collection of manuscripts to review and in offering comments and edits for the draft manuscript.

## Disclaimer

The views expressed in this manuscript are those of the authors and do not necessarily represent the views or policies of the United States Environmental Protection Agency. Any mention of trade names, products, or services does not imply an endorsement by the United States Government or the United States Environmental Protection Agency. The EPA does not endorse any commercial products, services, or enterprises.

## Conflict of Interest Statement

The authors declare that the research was conducted in the absence of any commercial or financial relationships that could be construed as a potential conflict of interest.
