# Supplementary material for: Ecotherapy – A Forgotten Ecosystem Service: A Review
Source: Front Psychol. 2018 Aug 3;9:1389. doi: 10.3389/fpsyg.2018.01389 (PMC6085576; doi:10.3389/fpsyg.2018.01389)
Supplement: Supplementary file 1 [file Table_1.DOCX]

Table S-1. Overview of reviewed studies of impact of interaction with nature and human condition change examining the nature of the change

Study Type Specific Study Reference Associational Weight-of-Evidence Holistic Hypothesis-Testing

Medical Recovery View Impact on Ulrich et al. 1991 X Recovery Rates

Flowers Impact on Park and Mattson 2008 X

Post Analgesic Rates

Ornamentals Impact Park and Mattson 2009a X

On Recovery Rates

Foliage Impact on Park and Mattson 2009b X

Recovery Rates

Foliage Impact on Raanaas et al. 2010 X

Well-being during

Rehabilitation

Review Plant Impacts - Bringslimark et al. 2009 X X X X Variety of Psychological

Outcomes

Horticultural Therapy Wichrowski et al. 2005 X

Impact on Cardio-

Pulmonary Recovery

Pain Reduction Review of Environment Malenbaum et al. 2008 X X X

Impact on Pain Control

Landscape View Impact Ulrich et al. 1993 X

on Pain Reduction

Table S-1. Overview of reviewed studies of impact of interaction with nature and human condition change examining the nature of the change

Study Type Specific Study Reference Associational Weight-of-Evidence Holistic Hypothesis-Testing

Pain Reduction Nature Images Impact Diette et al. 2003 X

On Pain Reduction

Nature Scenery Impact TSE et al. 2002 X

On Pain Tolerance

Sunlight Impact on Walch et al. 2005 X Analgesic Medication

Use

Plant Impacts on Park et al. 2002 X

Pain Tolerance

Plant Impacts on Lohr and Pearson- X X

Pain Perception Mims 2000

PTSD Recreation-Based Hawkins et al. 2016 X X X

Ecotherapy on PTSD

River Running Impact Dustin et al. 2011 X X

on PTSD

Fly-Fishing Impact on Mowatt and Bennett X

PTSD 2011

Mood Modification Therapeutic Recreation Anderson and Heyne X X

Practice Impact on 2012

Well-Being

Table S-1. Overview of reviewed studies of impact of interaction with nature and human condition change examining the nature of the change

Study Type Specific Study Reference Associational Weight-of-Evidence Holistic Hypothesis-Testing

Mood Modification Therapeutic Recreation Heyne and Anderson X X X

Strengths-Based 2012

Practice on Well-Being

Adventure Education Hattie et al. 1997 X X

Impact on Self-Concept

And Locus of Control

Wilderness Experience Fredrickson and X X

Impact on Spiritual Anderson 1999

Inspiration

Impact of Outdoor Ewert et al. 2001 X X X

Experiences on

Therapeutic Recreation

Green Space Impact Burls 2007 X X

On Public Health and

Mental Health

Group Behavior Sibthorp and Jostad X

Change Resulting from 2014

Outdoor Adventures

Horticultural Therapy Son et al. 2004 X X

Impact on Schizophrenia

Table S-1. Overview of reviewed studies of impact of interaction with nature and human condition change examining the nature of the change

Study Type Specific Study Reference Associational Weight-of-Evidence Holistic Hypothesis-Testing

Behavioral Wilderness Therapy Russell et al. 1999 X

Modification Impact on Adolescent

Behavioral Problems

Wilderness Therapy Hill 2007 X

Impact on Treatment

Of At-Risk Youth

Nature-assisted Annerstedt and X X X

Therapy Impact on Wahrborg 2011

Behavior

Wilderness Challenges Wilson and Lipsey X X

Impact on Delinquent 2000

Behavior

Wilderness Treatment Harper and Russell X X X

Impact on Adolescent 2008

Behavior

Wilderness Therapy Harper et al. 2007 X X

Impact on Adolescent

Behavior

Wilderness Treatment Bettman 2007 X

Impact on Adolescent

Attachment

Table S-1. Overview of reviewed studies of impact of interaction with nature and human condition change examining the nature of the change

Study Type Specific Study Reference Associational Weight-of-Evidence Holistic Hypothesis-Testing

Behavioral Wilderness Family Bandoroff and Schrer X

Modification Therapy Impacts on 1994

Adolescent Problems

Wander Gardens Detweiler et al. 2008 X

Impact of Dementia

Behaviors

Environmental Cimprich and Ronis X

To Restore Attention 2003

Nature Interaction to Cimprich 1992 X

Restore Attention

Nature Interaction to Cimprich 1993 X

Restore Attention

Nature Views Impact Tennessen and Cimprich X X

On Attention 1995

Environment Impacts Kuo 2001 X X

On Attention in

Inner City

Nature Views Impacts Faber Taylor et al. 2002 X

On Self-Discipline

Longevity Walkable Green Space Takano et al. 2002 X

Impact on Senior

Citizens

Table S-1. Overview of reviewed studies of impact of interaction with nature and human condition change examining the nature of the change

Study Type Specific Study Reference Associational Weight-of-Evidence Holistic Hypothesis-Testing

Disease Treatment Lifestyle Change Ornish et al. 1990 X

Impact on

Atherosclerosis

Psychological & Social Krantz and McCeney X X X

Factors Impact on 2002

Organic Disease

Garden and Art McCafferey 2007 X

Therapy Impacts

on Depression

Indoor Gardening Lee and Kim 2008 X X

Impact on Dementia

Horticultural Therapy Lee et al. 2008 X

Impact on Depression

Horticultural Therapy Gigliotti et al 2004 X

Impact on Dementia

Horticultural Therapy Gigliotti and Jarrott X X

Impact on Dementia 2005

Adventure-Based Voruganti et al. 2006 X

Impact on Schizophrenia

Nature Therapy and Chalfont 2007 X

Dementia

Table S-1. Overview of reviewed studies of impact of interaction with nature and human condition change examining the nature of the change

Study Type Specific Study Reference Associational Weight-of-Evidence Holistic Hypothesis-Testing

Disease Treatment Environmental Therapy Day et al. 2000 X X X X

Impacts on Dementia

Outdoor Spaces Cobley 2002 X

Impacts on Dementia

Outdoor Space Impact Lovering 1990 X

On Alzheimer’s Disease

Garden Space Impact Mather et al. 1997 X

On Alzheimer’s Disease

Sunlight and Vitamin D Holick 2004 X

Impacts on Disease

Outdoor Activities Kampman et al. 2007 X

Impact on Multiple

Sclerosis

Substance Abuse Horticultural Therapy Richards and Kafami X

Impact on Substance 1999

Abuse

Therapeutic Camping Bennett et al. 1998 X X

Impact on Substance

Abuse

Outward Bound Kennedy and Minami X X

Impact on Substance 1993

Abuse

Table S-1. Overview of reviewed studies of impact of interaction with nature and human condition change examining the nature of the change

Study Type Specific Study Reference Associational Weight-of-Evidence Holistic Hypothesis-Testing

Coping Skills Adventure Camp Banaka and Young X

Impact on Coping Skills 1985

Self Worth Adventure Course Luckner 1989a X X

Impacts on Self-Concept

Horticultural & Abbott et al. 2007 X

Recreational Therapy

Impacts on Elderly

Self-worth

Nature-based Activities van Loon 2004 X

Impacts on Elderly

Locus of Control Outdoor Adventure Luckner 1989b X X

Impact of Locus of

Control in Hearing

Impaired

Wilderness Therapy Davis-Berman and Berman X

Impact on Adolescent 1989

Locus of Control

Brain Injury Outdoor Experience Thomas 2004 X X

Adjustment Impact on Brain

Injury Adjustment

Well Being Wilderness Therapy Harper and Cooley X X

Impact on Adolescent 2007

& Family Well-Being

Table S-1. Overview of reviewed studies of impact of interaction with nature and human condition change examining the nature of the change

Study Type Specific Study Reference Associational Weight-of-Evidence Holistic Hypothesis-Testing

Well-Being Horticulture Impacts Relf 1992 X X X

On Well Being

Exposure to Nature Bowler et al. 2010 X X

Impact on Well-Being

Green Exercise Impact Pretty et al. 2003 X

On Well-being

Green Exercise Impact Pretty et al. 2005 X X

On Well-being

Green Exercise Impact Pretty et al. 2007 X X

On Well-being

Green Exercise Impact Peacock et al. 2007 X X

On Well-being

Nature Exposure Bird 2007 X

Impact on Well-Being

Greenspace Impact Burls 2007 X X

On Well-Being

Nature Exposure MIND 2007 X X

Impact on Well-Being

Outdoor Running McMurray et al. 1988 X X

On Blood Chemistry

And Well-being

Table S-1. Overview of reviewed studies of impact of interaction with nature and human condition change examining the nature of the change

Study Type Specific Study Reference Associational Weight-of-Evidence Holistic Hypothesis-Testing

Well-Being Outdoor Running Harte and Eifert 1995 X X

On Blood Chemistry

And Well-being

Greenspace Barton et al. 2009 X

Benefits to Well-being

Walking in Nature Focht 2009 X X

Impacts on Well-being

Nature-Based Greenleaf et al. 2014 X X

Counselling Impacts

On Well-Being

Stress Reduction Garden Walk Impacts Kohlleppel and Bradley X

On Stress 2002

Nature Buffers and Wells and Evans 2003 X X

Impact on Stress

Natural Settings Impact Hartig et al. 2003 X

On Stress Reduction &

Attention Restoration

Virtual Reality and Plante et al. 2003 X X

Nature Exposure Impact

On Stress

Table S-1. Overview of reviewed studies of impact of interaction with nature and human condition change examining the nature of the change

Study Type Specific Study Reference Associational Weight-of-Evidence Holistic Hypothesis-Testing

Stress Reduction Virtual Reality and Plante et al. 2006 X X

Nature Exposure Impact

On Stress

Virtual Reality and Plante et al. 2007 X X

Nature Exposure Impact

On Stress

Outdoor Exercise Kerr et al. 2006 X X

Impact on Stress

Walking in Nature Teas et al. 2007 X X

Impacts on Well-being

Child Development Green Space Impact Faber Taylor et al. 1998 X X

On Creativity

Natural Playscape Fjortoft and Sageie 2000 X

Impact on Learning &

Development

Outdoor Play Impact Fjortoft 2001 X

On Development

Natural Environment Fjortoft 2004 X X

Impacts on Motor

Development

Table S-1. Overview of reviewed studies of impact of interaction with nature and human condition change examining the nature of the change

Study Type Specific Study Reference Associational Weight-of-Evidence Holistic Hypothesis-Testing

Child Development Outdoor Spaces Karsten 2005 X

Impacts on Child

Development

Environmental Play Veitch et al. 2010 X X

Impact on Child

Development

Environmental Play Little and Wyver 2008 X X

Risk and Child

Development

Outdoor Wilderness Kellert and Derr 1998 X X X X

Experience Impacts on

Child Development

Experiencing Nature Kellert 2002 X X X X

Impacts on Child

Development

Nature Experiences Kahn 2002 X X X

Impacts on Child

Development

Outdoor Summer Kaplan 1977 X X X

Programs Impacts on

Child Development

Table S-1. Overview of reviewed studies of impact of interaction with nature and human condition change examining the nature of the change

Study Type Specific Study Reference Associational Weight-of-Evidence Holistic Hypothesis-Testing

Child Development Nature Benefits for Kaplan and Talbot 1983 X

Child Development

Environmental Ratanapojnard 2001 X

Education Impacts

On Child Development

Natural Setting Impact Kirkby 1989 X

On Child Development

ADHD Natural Setting Effect van den Berg and van den X X

On ADHD Children Berg 2010

Green Play Setting Taylor et al. 2001 X

Impacts on ADD

Green Setting Impacts Kuo and Faber Taylor 2004 X X

On ADHD

Green Space Walk Faber Taylor and Kuo 2009 X X

Impacts on ADHD

Obesity Access to Nature Neslen 2017 X

Impacts on Obesity

& Depression

Greenspace Impact Lachowycz and Jones X X

On Obesity 2011

Table S-1. Overview of reviewed studies of impact of interaction with nature and human condition change examining the nature of the change

Study Type Specific Study Reference Associational Weight-of-Evidence Holistic Hypothesis-Testing

Obesity Green Neighborhoods Liu et al. 2007 X

Impact on Obesity

Access to Greenness Tilt et al. 2007 X X

Impact on Obesity

Greenness and Body Bell et al. 2008 X

Mass Index

Greenery Impacts on Ellaway et al. 2005 X X

Adult Obesity

Cognitive Function Foraging in Nature Chipeniuk 1995 X X

Impacts on Cognitive

Development

School Trips and Falk and Dierking X X

Cognitive Development 1997

Greenness Impacts Wells 2000 X X

On Cognitive Function

Science Field Trips & Kisiel 2005 X X

Cognitive Development

Nature Interactions Berman et al. 2008 X X X

Impact on Cognition

Public Health Green Infrastructure Tzoulas et al. 2007 X X X X

Impact on Public Health

Table S-1. Overview of reviewed studies of impact of interaction with nature and human condition change examining the nature of the change

Study Type Specific Study Reference Associational Weight-of-Evidence Holistic Hypothesis-Testing

Restoration Interaction with Kaplan and Kaplan X X X X

Nature Impact on 1989

Personal Restoration

Natural Environment Hartig et al. 1991 X X X

Experiences Impact on

Restoration

Nature Effects on Ryan et al. 2010 X X X

Revitalization

Forest Activity Hug et al. 2008 X X

Impacts on Restoration
